# Sporting participation following the operative management of chondral defects of the knee at mid-term follow up: a systematic review and meta-analysis

**DOI:** 10.1186/s40634-020-00295-x

**Published:** 2020-10-06

**Authors:** P. G. Robinson, T. Williamson, I. R. Murray, K. Al-Hourani, T. O. White

**Affiliations:** 1 Edinburgh Orthopaedics, Royal Infirmary of Edinburgh, Edinburgh, Scotland; 2 University of Edinburgh Medical School, Edinburgh, Scotland

**Keywords:** Biologics, Regeneration, Repair, Chondrocyte, Athlete

## Abstract

**Purpose:**

The purpose of this study was to perform a systematic review of the reparticipation in sport at mid-term follow up in athletes who underwent biologic treatment of chondral defects in the knee and compare the rates amongst different biologic procedures.

**Methods:**

A search of PubMed/Medline and Embase was performed in May 2020 in keeping with Preferred Reporting Items for Systematic Review and Meta-Analysis (PRISMA) guidelines. The criteria for inclusion were observational, published research articles studying the outcomes and rates of participation in sport following biologic treatments of the knee with a minimum mean/median follow up of 5 years. Interventions included microfracture, osteochondral autograft transfer (OAT), autologous chondrocyte implantation (ACI), matrix-induced autologous chondrocyte implantation (MACI), osteochondral allograft, or platelet rich plasma (PRP) and peripheral blood stem cells (PBSC). A random effects model of head-to-head evidence was used to determine rates of sporting participation following each intervention.

**Results:**

There were twenty-nine studies which met the inclusion criteria with a total of 1276 patients (67% male, 33% female). The mean age was 32.8 years (13–69, SD 5.7) and the mean follow up was 89 months (SD 42.4). The number of studies reporting OAT was 8 (27.6%), ACI was 6 (20.7%), MACI was 7 (24.1%), microfracture was 5 (17.2%), osteochondral allograft was 4 (13.8%), and one study (3.4%) reported on PRP and PBSC. The overall return to any level of sport was 80%, with 58.6% returning to preinjury levels. PRP and PBSC (100%) and OAT (84.4%) had the highest rates of sporting participation, followed by allograft (83.9%) and ACI (80.7%). The lowest rates of participation were seen following MACI (74%) and microfracture (64.2%).

**Conclusions:**

High rates of re-participation in sport are sustained for at least 5 years following biologic intervention for chondral injuries in the knee. Where possible, OAT should be considered as the treatment of choice when prolonged participation in sport is a priority for patients. However, MACI may achieve the highest probability of returning to the same pre-injury sporting level.

**Level of evidence:**

IV

## Background

Chondral defects in the knee are problematic for athletes primarily as a consequence of 1) focal defects having intrinsically a poor ability to heal and 2) the early development of symptomatic arthritis. These injuries are reported to be 20% more common in athletes compared to the general population [2] and can lead to lifestyle modification (such as ceasing sporting participation) due to pain and loss of function. Therefore, it is imperative that tailored and effective treatment options are offered to these patients. Ideally, such treatments will preserve the integrity of the native knee and return the patient to their pre-injury performance level within an acceptable timeframe. Patients most at risk of chondral injuries include those with a higher body mass index (BMI) or who have previously had an anterior cruciate ligament (ACL) injury [1, 15]. Chondral injury has been reported to present in up to 50% of patients undergoing ACL reconstructive surgery [4, 6].

The current treatment options available for the management of focal chondral defects include microfracture, autologous chondrocyte implantation (ACI), matrix-induced autologous chondrogenesis (MACI), osteochondral autograft/allograft implantation and injectable cell-based therapies. Two previous meta-analyses and a systematic review have analysed the rates of return to sport (RTS) following surgical management of articular knee defects with a range from 65% to 78% at mean follow up of 42 and 47 months respectively [19, 24]. Although RTS is considered one of the most important outcome measures for athletes following injury, there is little summarised information regarding the sustained rate of participation in sport following biologic interventions for the knee.

The purpose of this study was to perform a systematic review of the mid-term outcomes and rates of re-participation in sport in athletes who underwent biologic intervention of chondral knee defects.

## Methods

A search of PubMed/Medline and Embase was performed in May 2020 in line with the Preferred Reporting Items for Systematic Review and Meta-Analysis (PRISMA) guidelines. The study was registered using the PROSPERO International prospective register of systematic reviews. The search terms used were: ‘knee AND (chondral OR chondrocyte OR osteochondral or cartilage) AND (allograft OR autograft OR autologous OR implantation OR transplantation OR mosaicplasty OR OAT OR OATS OR microfracture OR MACI OR ACI OR platelet OR plasma OR mesenchymal OR stem OR BMAC OR bone marrow OR concentrate) AND (sport OR athlete)’.

Inclusion criteria were research articles studying the use of biologic techniques to repair or regenerate knee cartilage in athletes. Studies were included if the mean or median follow up was a minimum of 5 years. Studies were excluded if they had a mean/median follow up of less than 5 years, did not report on postoperative return to sport rates, were review articles, editorials/letters to the editor, or were not published in the English language.

**Fig. 1:**
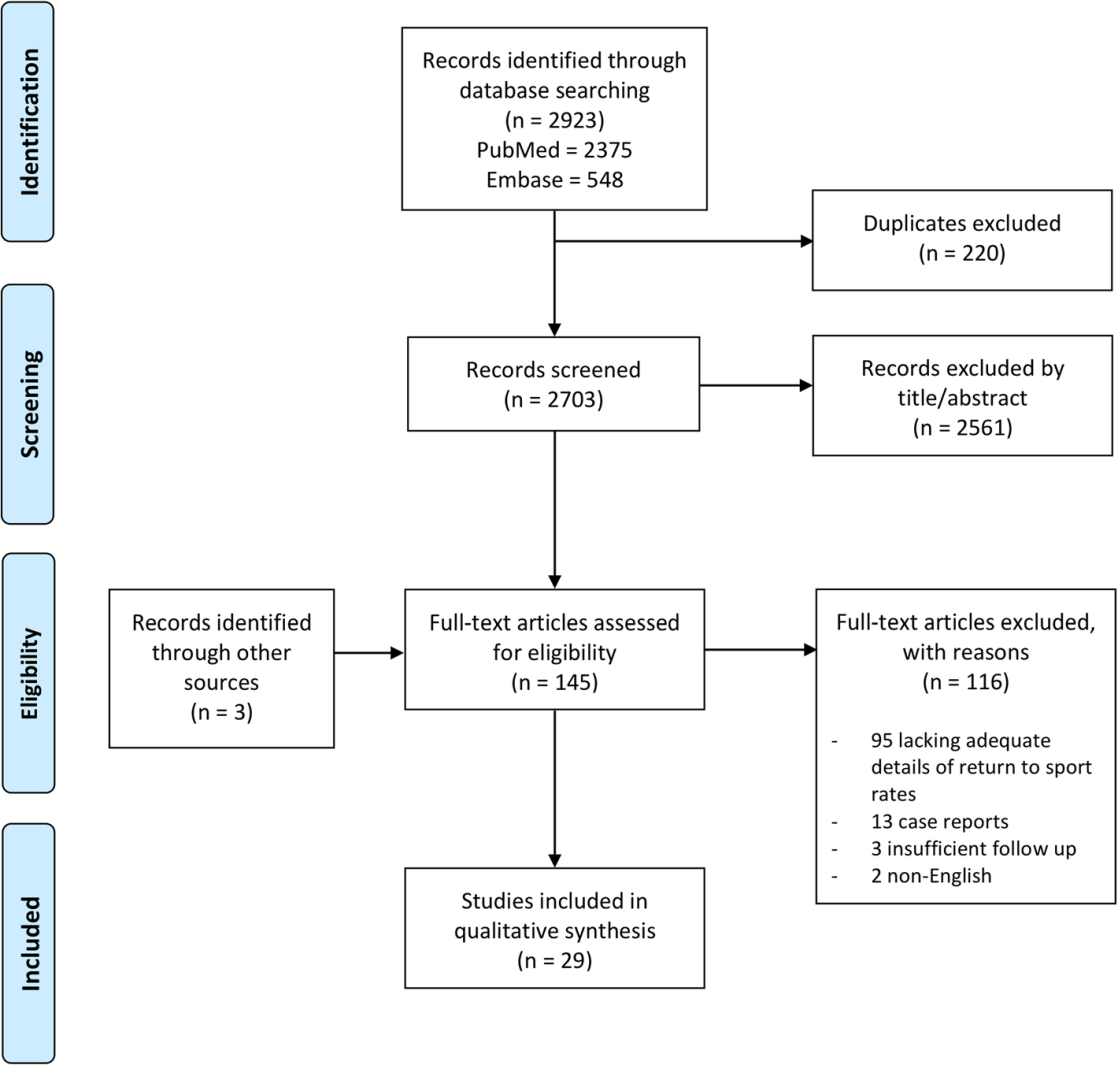
PRISMA flow diagram showing the study selection process

Titles and abstracts identified were independently reviewed by two authors (P.G.R., T.R.W) and those not meeting the inclusion criteria were excluded prior to full text review. Full text studies were further evaluated against the inclusion and exclusion criteria. The references of the included papers were also reviewed to ensure no relevant studies were missed. The search process is presented in Fig. 1.

The year of publication, age, gender, BMI, size of chondral lesion, lesion location, type of biologic, rate of return to sport, time to return, level of participation and postoperative patient reported outcome measures were recorded, if available, for each study.

### Outcome measures

Our primary outcome was the rate of reparticipation in sport in athletes undergoing biologic knee surgery at mid-term follow up. Secondary outcomes included comparing the time to RTS, level of sport the athlete returned to and patient reported outcome measure scores (PROMS) at mid-term follow up. A meta-analysis of the primary outcome by each biologic intervention was performed if the studies were sufficiently homogenous.

### Quality assessment

All studies were assessed for quality by two authors (P.G.R., T.W.) using the National Institute of Health Quality Assessment Tool for Observational Cohort and Cross-Sectional Studies. The assessment tool uses 14 questions to enable allocation of a score to each article (poor, fair or good). If there was disagreement regarding the scoring of a study, consensus will be met after discussion amongst both assessors.

### Statistical analysis

Statistical analysis was performed using Statistical Package for Social Sciences (SPSS) software (IBM, Inc., Armonk, New York, United States) v24. Data were tested for parametricity using Kolmogorov-Smirnov testing. Continuous variables were reported by means and standard deviations and compared using the student T test and categorical data was compared the using chi-squared test. Nonparametric tests (Kruskal-Wallis and Mann-Whitney U tests) were used to assess for differences in sporting participation rates between interventions. Correlation between continuous variables and rates of sporting participation were assessed using Pearson’s coefficient. Heterogeneity between studies was tested using preoperative parameters of age, BMI, lesion size and preoperative outcome measure score using *I*^*2*^ index based on Cochran’s Q with an *I*^*2*^ index greater than 50% deemed heterogenous. Random effects modelling was used to measure effect size of the type of biologic and rate of participation in sport. The data was standardised to means and standard deviations (SDs), weighted for sample size.

## Results

There were 2703 articles identified in the initial search of databases. After initial screening of titles and abstracts, 142 articles met the inclusion criteria for review. On full text screening, a further 113 studies were excluded as they met the exclusion criteria (Fig. 1). There were twenty-nine studies which met the inclusion criteria (Table 1). The year of publication ranged from 2004 to 2019. There were 24 case series, two prospective cohort studies, 2 randomised controlled trials and one case-control study. There were a total of 1276 patients included across all studies. The mean age was 32.8 years (± 5.7) and 67% were males and 33% females. The mean length of follow up was 88.8 months (± 42.4). Patient demographics can be seen in Table 2.

**Table 1:** Patient demographics and outcome measures of included studies

Study	Journal	Year	LOE	No. of patients	Gender	Mean age (range)	Follow up (months)	Outcome measures
Cotter et al. [3]	Arthroscopy	2018	IV	22	NR	24.39 (NR)	87	RTS
Ebert et al. [7]	American Journal of Sports Medicine	2015	IV	31	15 M, 16 F	35.3 (16–57)	60	RTS, KOOS, Lysholm, Tegner, SF-36
Ebert et al. [8]	American Journal of Sports Medicine	2012	I	63	42 M, 21 F	38.2 (16–63)	60	RTS, KOOS, SF-36
Ebert et al. [9]	American Journal of Sports Medicine	2011	IV	41	21 M, 20 F	38.5 (13–65)	60	RTS, KOOS, SF-36
Ebert et al. [10]	Orthopaedic Journal of Sports Medicine	2019	III	97	60 M, 37 F	36.8 (15–62)	60	RTS, KOOS
Gillogly et al. [11]	American Journal of Sports Medicine	2014	IV	23	11 M, 12 F	31 (NR)	91	RTS, IKDC, Modified Cincinnati, Lysholm, SF-12
Gobbi et al. [12]	Knee Surgery Sports Traumatology & Arthroscopy	2014	IV	61	43 M, 18 F	31.4 (NR)	181	RTS, IKDC, Lysholm, Tegner
Gobbi et al. [13]	Knee Surgery Sports Traumatology & Arthroscopy	2005	IV	53	38 M, 33 F	38 (19–55)	72	RTS, IKDC, Lysholm, Tegner
Gudas et al. [14]	American Journal of Sports Medicine	2012	I	57	36 M, 21 F	24.3 (15–40)	125	RTS, ICRS, Tegner
Kon et al. [16]	American Journal of Sports Medicine	2011	II	21	21 M	23.7 (16–37)	94	RTS, IKDC, EQ-VAS,
Kon et al. [18]	American Journal of Sports Medicine	2009	II	80	60 M, 20 F	29.8 (NR)	60	RTS, IKDC, ICRS, Tegner
Liu et al. [20]	Arthroscopy	2019	IV	13	NR	NR	112	RTS, Marx
Marcacci et al. [21]	American Journal of Sports Medicine	2007	IV	30	22 M, 8 F	29.3 (17–46)	84	RTS, IKDC, ICRS, Tegner
McCarthy et al. [22]	Arthroscopy	2017	IV	13	7 M, 6 F	19.2 (15.4–26.1)	71	RTS, IKDC, KOOS, WOMAC, SF-12, Marx, Tegner
Minzlaff et al. [23]	Knee Surgery Sports Traumatology & Arthroscopy	2016	IV	30	NR	31 (19–39)	83	RTS, Activity rating scale, Tegner
Monckeberg et al. [25]	Knee	2019	IV	20	13 M, 7 F	32.7 (21–47)	60	RTS, IKDC
Nielsen et al. [26]	American Journal of Sports Medicine	2017	IV	149	87 M, 62 F	31.2 (NR)	72	RTS, Merle d’Aubigne´-Postel score, IKDC, Knee Society Function Score
Ollat et al. [27]	Orthopaedics & Traumatology: Surgery & Research	2011	IV	20	NR	NR	96	RTS
Panics et al. [28]	Cartilage	2012	IV	61	55 M, 6 F	25.2 (16–41)	115	RTS, ICRS, HSS, Mod-Cincinnati, Lysholm
Pelissier et al. [29]	Knee Surgery Sports Traumatology & Arthroscopy	2014	IV	12	10 M, 2 F	29 (NR)	120	RTS, IKDC, Lysholm
Pestka et al. [30]	American Journal of Sports Medicine	2016	IV	130	81 M, 49 F	36.2 (14.8–52.9)	64	RTS, Tegner
Ronga et al. [31]	Joints	2015	IV	4	3 M, 1 F	21.2 (18–24)	126	RTS, Mod Cincinnati, Lysholm, Tegner
Scillia et al. [32]	American Journal of Sports Medicine	2015	IV	18	18 M	NR	71	RTS
Stone et al. [33]	Knee Surgery Sports Traumatology & Arthroscopy	2017	IV	74	46 M, 28 F	45.3 (13–69)	202	RTS, IKDC, WOMAC, Tegner
Tetta et al. [34]	European Journal of Radiology	2010	IV	24	17 M, 7 F	29.9 (NR)	113	RTS, IKDC, Tegner
Vijayan et al. [35]	The Journal of Bone and joint Surgery (British)	2012	IV	14	12 M, 2 F	23.6 (16–40)	62	RTS, Mod-Cincinnati, Stanmore-Bentley
Viste et al. [36]	Orthopaedics & Traumatology: Surgery & Research	2012	IV	14	7 M, 7 F	37.7 (30–45)	72	RTS, IKDC, Brittberg-Peterson,
Zaffagnini et al. [37]	Knee Surgery Sports Traumatology & Arthroscopy	2019	IV	31	31 M	22.6 (NR)	120	RTS, IKDC, Tegner
Zak et al. [38]	American Journal of Sports Medicine	2012	IV	70	51 M, 19 F	34.9 (18–55)	60	RTS, KOOS, Noyes, Tegner

*LOE* level of evidence, *NR* not reported, *M* male, *F* female, *No* number, *RTS* return to sport, *KOOS* knee injury and osteoarthritis outcome score, *SF-36* short-form-36 health survey questionnaire, *SF-12* short-form-12 health survey questionnaire, *IKDC* international knee documentation committee, *ICRS* international cartilage repair society, *EQ-VAS* EuroQuol visual analogue scale, *WOMAC* western Ontario and McMaster universities osteoarthritis index, *HSS* hospital for special surgery, *Mod* modified

**Table 2:** Patient demographics from the included studies

	MFX	ACI	MACI	OAT	Allograft	PRP + PBSC	Overall
Studies	5	6	7	8	4	1	29^a^
Patients (n)	201	240	347	271	197	20	1276
Age in years (SD)	32.0 (± 4.5)	33.1 (± 4.3)	34.9 (± 4.8)	32.6 (± 8.5)	29.5 (± 3.6)	32.7(± 7.5)	32.8 (± 5.7)
Gender (M/F, %)	69/31	68/32	67/33	73/27	58/42	65/35	67/33
BMI	NR	24.3 (± 0.5)	26.1 (± 0.8)	25.0 (± 2.5)	25.0 (± 0.6)	26.0 (± 2.7)	25.3 (± 1.0)
Follow up in months (SD)	114.1 (± 51.8)	71.8 (± 15.9)	65.5 (± 17.1)	136.5 (± 47.9)	71.9 (± 0.3)	61.2 (NR)	88.8 (± 42.4)
Preoperative duration of symptoms in months (SD)	NR	32.9 (± 5.1)	94.6 (± 15.3)	NR	58.4 (± 1.0)	4.3 (NR)	71.9 (± 28.1)
Preoperative mean Tegner score (SD)	3.2 (NR)	3.50 (± 1.3)	1.9 (± 0.5)	2.9 (± 0.0)	NR	NR	2.5 (± 0.7)
Preoperative mean Lysholm score (SD)	50.7 (± 5.7)	40.5 (± 0.4)	53.8 (± 6.9)	64.7 (± 5.2)	41.0 (± 13.0)	NR	52.7 (± 9.3)
Preoperative mean IKDC subjective score (SD)	44.5 (± 2.8)	41.3 (1.4)	40.3 (± 13.4)	34.8 (± 13.5)	38.0 (± 12.0)	50.5 (± 6.3)	42.1 (± 4.0)
Chondral defects							
Size in cm^2^ (SD)	3.5 (± 0.7)	4.0 (± 1.4)	3.6 (± 1.2)	2.4 (± 0.5)	4.5 (± 1.8)	NR	3.4 (± 1.2)
MFC (n)	99	120	187	155	9	NR	570
LFC (n)	37	37	74	54	6	NR	217
Trochlea (n)	13	18	14	16	21	9	90
Patella (n)	7	68	11	4	12	6	108

*NR* not reported; *MFX* microfracture, *ACI* autologous chondrocyte implantation, *MACI* matrix-induced autologous chondrocyte implantation, *OAT* osteochondral autograft transfer, *PRP* platelet-rich plasma, *PBSC* peripheral blood stem cells, *M* male, *F* female, *IKDC* international knee documentation committee, *MFC* medial femoral condyle, *LFC* lateral femoral condyle. ^a^Two studies compared more than one biologic technique

### Quality assessment

The results of the quality assessment of included studies can be seen in Appendix 1. The overall mean percentage of successfully answered questions from the assessment tool was 61.1% (range 42.9% to 85.7%). Two studies were level one, two were level two, one was level three, and twenty-four were level four.

### Return to sport

**Fig. 2:**
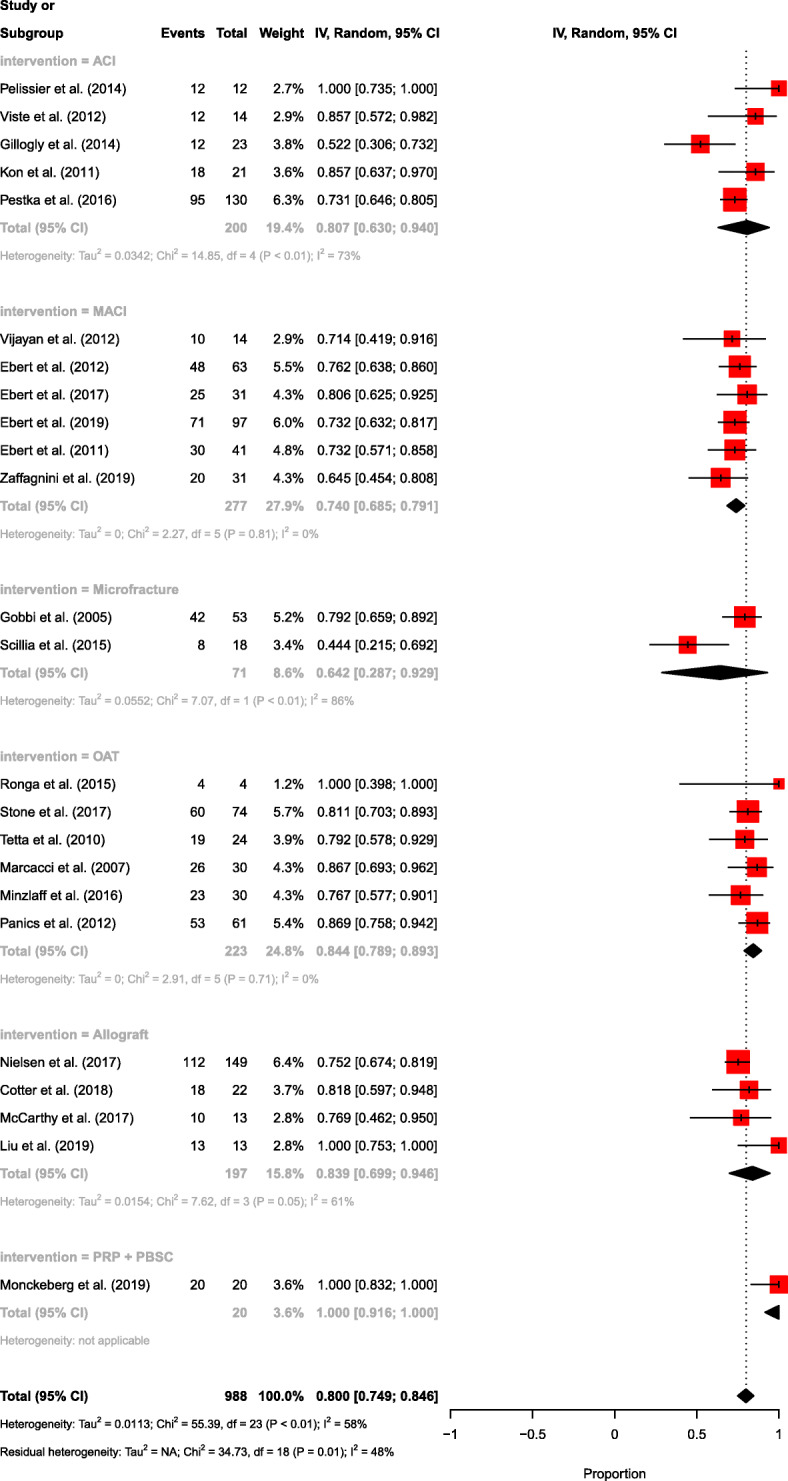
Forest plot comparing each surgical intervention and the rate of returning to sport

**Fig. 3:**
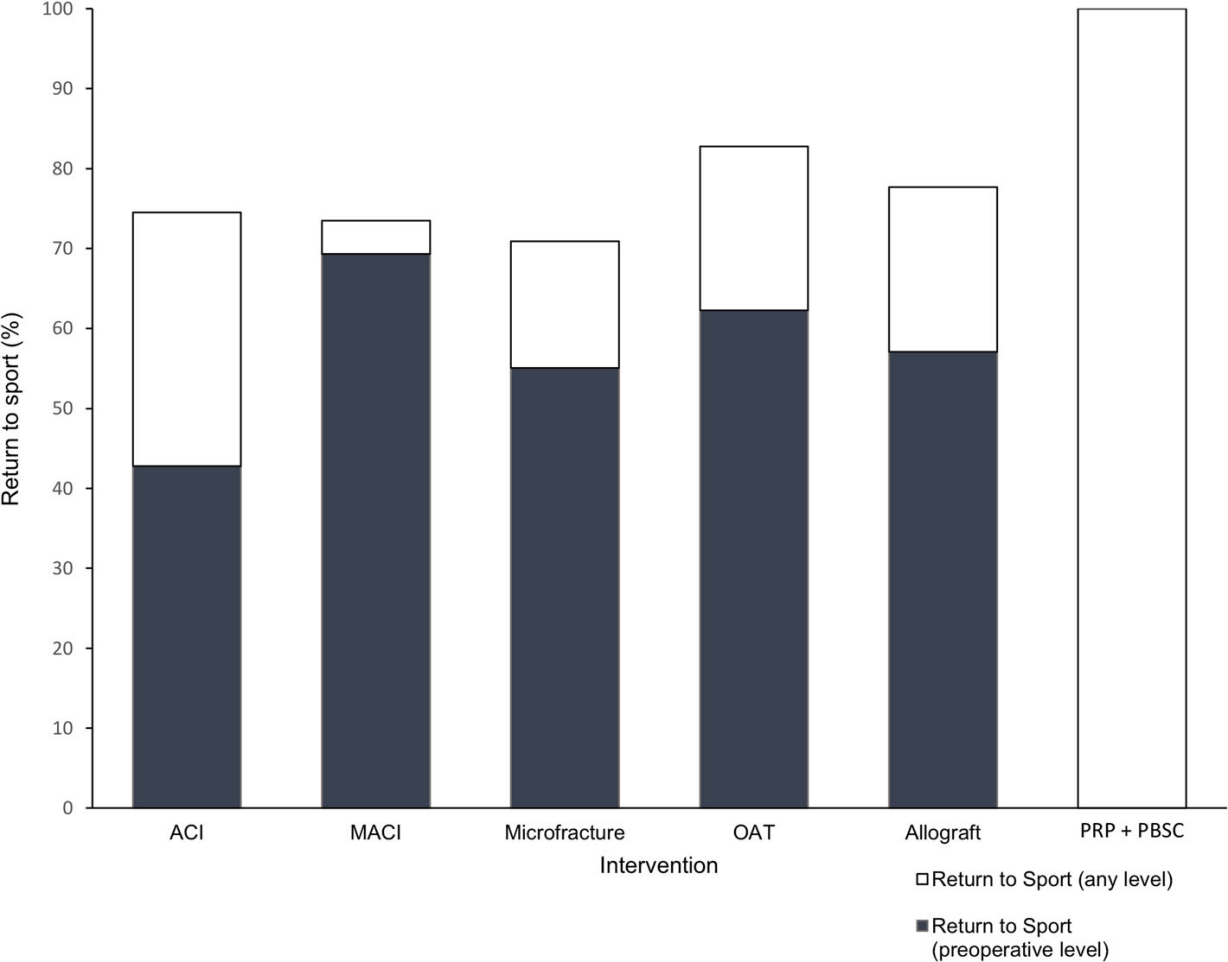
Proportion of patients returning to sport and returning to the same preoperative level of sport

The overall rate of patients participating in sport at any level was 80.0% (Fig. 2). Sporting participation following microfracture was 64.2%, MACI was 74.0%, ACI was 80.7%, Osteochondral allograft was 83.9%, OAT was 84.4%, and PRP and PBSC was 100% (*p* < 0.001). There were sixteen studies which reported on the rate of returning to preinjury sports levels, with 58.6% returning to their preinjury performance. Rates of return to preinjury levels of sport were highest following MACI (69.3%), followed by OAT (62.3%), osteochondral allograft (57.1%) and microfracture (55.1%), and lowest rates were seen following ACI (42.7%) (*p* < 0.001) (Fig. 3).

There were eight studies which reported the mean time to return to sports. The mean time to return to sport was 7.4 months (± 2.9), although this was only reported for OAT (four studies), allograft (three studies), and microfracture (one study).

### Secondary outcomes

#### Patient reported outcome measures

At final follow up the mean Lysholm score was 84.4% (± 7.6) and had a weak, positive correlation with return to sport rate (*r* = 0.52, *p* < 0.001). The highest Lysholm scores were seen following OAT (93.2% ± 0.9), followed by MACI (86.8% ± 4.2), microfracture (81.85% ± 5.0), ACI (81.6 ± 3.2), and osteochondral allograft (64.0% ± 18). The overall mean Tegner score at final follow up was 4.84 (± 1.37). The mean Tegner scores were 6.1 (± 0.5) following OAT, 5.4 (± 0.5) following microfracture, 4.9 (± 1.2) following MACI, 4.5 (± 2.1) following osteochondral allograft, and 4.0 (± 1.5) following ACI. There was a weak, positive correlation between rate of return to sport and mean Tegner score at final follow up (*r* = 0.336, *p* < 0.001). Mean IKDC score overall was 75.2. Highest scores were observed following MACI (87.3 ± 13.6), followed by ACI (78.5 ± 8.7), PRP and PBSC (72.2 ± 13.3), OAT (71.7 ± 18.8), microfracture (71.0 ± 0.6), and osteochondral allograft (63.0 ± 22.0). There was no correlation between return to sport and either IKDC or KOOS scores (*r* = − 0.238, *p* = 0.002; and *r* = 0.179, *p* = 0.005 respectively).

#### Previous & concurrent operations

There were 64.3% of patients who had undergone at least one previous surgery on their affected knee prior to the current biologic intervention. This was highest in those undergoing osteochondral allograft (88.6%), whilst patients undergoing microfracture had the lowest proportion of previous surgeries (25%). Concomitant procedures were performed alongside 35.6% of the operations. This was highest in ACI (69.0%) and OAT (66.4%). No patients in the PRP and PBSC group underwent any previous or additional procedures.

#### Lesion size

The mean size of chondral lesions was reported in 22 studies and the mean lesion area was 3.4cm^2^ (± 1.2) (Table 2). A weak, negative correlation between lesion size and return to any level of sport was observed (*r* = − 0.408, *p* < 0.001). No significant differences in lesion size between intervention groups were observed (*p* = 0.131).

## Discussion

This is the first systematic review and meta-analysis to assess the post-operative sporting participation in patients who have undergone biologic intervention for chondral knee defects at mid-term follow up. There is a high rate of sustained return to sport observed following surgery, with relatively worse outcomes for those who have undergone microfracture compared to other modes of intervention.

The sporting participation rate of 80% is similar to a previous meta-analysis of return to sport following biologic chondral intervention, which found a 76% return to sport at a much shorter mean follow up of 47 months [19]. Mithoefer et al. also performed a similar meta-analysis with mean follow up of 42 months and found rates of return to sport of 79% [24]. Our findings that sporting participation was highest following OAT and lowest following microfracture also align with those by Krych et al [19], although the authors found higher rates of returning sport in their study following OAT compared to the present study (92.9% vs 84.4%) albeit with shorter follow up. Krych et al found slightly lower rates of returning to sport following microfracture compared to the current study (57.6% and 64.2% respectively). Mithoefer et al. also found microfracture to yield the worst rates of returning to sport (68%) [24]. We additionally reported individual return to sport rates following ACI and MACI. We showed ACI to have higher rates of sporting participation (80.7%) compared to MACI (74.0%, *p* = 0.017). However, more patients returned to preoperative performance levels following MACI compared to ACI (69.3% vs 42.7%, *p* < 0.001).

The highest rates of sporting participation were seen following OAT (82.8%) with 62.3% of patients returning to their preoperative sports performance. Microfracture resulted in the worst rates of return to sport with 64.2%, which was substantially lower than other treatment options. Only one study reported on PRP and PBSC, with all 20 of its patients returning to sport, although it did not specify to which level. MACI reported the highest rate of return to the same preoperative sports level (69.3%). Overall, almost half of patients in this study were not continuing to participate at the same level of sport. However, with a mean age of 32.8 and a mean follow up of 7.4 years this decline of sporting activity may be explained by increasing age. This is the first systematic review to analyse the level of sport that athletes had returned to at mid-term follow up after chondral surgery of the knee. In contrast, Gobbi et al. [13] found that their observed decrease in return to sport was more likely in those with larger or multiple cartilage lesions, suggesting that knee-related factors may also play a role. Additionally, Gudas et al. found a significantly greater decrease in return to sport rates during the postoperative period following microfracture than following OAT [14]. Kon et al. reported similar findings with a decrease in long-term sports participation in their microfracture group in comparison with ACI [17].

The primary limitations of this review were regarding the heterogeneity of the literature and poor quality of some level four studies. Studies reported on a variety of different sports, with varying functional demands. This variation could account, in part, for differences in rates of return to sport. Additionally, there was no common definition for what constituted a return to sport; most papers analysed a return to any level of sport, but some exclusively assessed attainment of preoperative performance levels. Since the sporting objectives for the studies assessing return to preoperative levels of sport were not comparable to those reporting on return to any level, these papers were analysed separately. Some studies assessed professional athletes, some amateur, and some had a mixed population. This difference may account for variation in return to sport rates, particularly regarding return to preoperative performance level.

Furthermore, despite the influence rehabilitation has on the outcomes of cartilage preserving surgery within the knee [5], we could not take this into consideration in the random effects model analysis due to heterogenous reporting of protocols amongst studies. Finally, there was some variation in lesion size amongst the groups, however this was not statistically significant. OAT was typically used for smaller sized lesions, which may reflect a less severe injury to the knee and lead to faster and improved rates of return to sport. Concomitant procedures and previous surgeries were also confounding variables which may have affected patient outcome, for example a simultaneous ACL reconstruction. In contrast, the study measuring PRP and PBSC reported no concomitant procedures which may reflect a more benign knee injury and hence high levels of sporting participation postoperatively.

## Conclusion

A substantial number of patients continue to participate in sport at long term follow up following biologic interventions for chondral defects of the knee. Surgeons should be aware of the poorer rates of sporting participation at mid-term when using microfracture. OAT should be considered for athletes seeking to benefit from sustained future involvement in sport while MACI may achieve the highest probability of returning to the same level of sport prior to injury.

## Supplementary information

**Additional file 1.**

## Data Availability

All data generated or analysed during this study are included in this published article [and its supplementary information files].

## Abbreviations

NR: Not reported
MFX: Microfracture
ACI: Autologous chondrocyte implantation
MACI: Matrix-induced autologous chondrocyte implantation
OAT: Osteochondral autograft transfer
PRP: Platelet-rich plasma
PBSC: Peripheral blood stem cells
KOOS: Knee injury and osteoarthritis outcome score
SF-36: Short form 36
IKDC: International knee documentation committee
SF-12: Short form 12
EQ-VAS: EQ visual analogue scale
WOMAC: Western Ontario and McMaster universities osteoarthritis index
