# Supplementary material for: Sporting participation following the operative management of chondral defects of the knee at mid-term follow up: a systematic review and meta-analysis
Source: J Exp Orthop. 2020 Oct 6;7:76. doi: 10.1186/s40634-020-00295-x (PMC7538489; doi:10.1186/s40634-020-00295-x)
Supplement: Supplementary file 1 — Additional file 1. [file 40634_2020_295_MOESM1_ESM.pdf]

Criteria	Zak et al.	Panics et al.	Marcacci et al.	Ebert et al. 2011	Ebert et al. 2017	Ebert et al. 2012	Ebert et al. 2019	Kon et al. 2009	Kon et al. 2011
1. Was the research question or objective in this paper clearly stated?	Y	Y	Y	Y	Y	Y	Y	Y	Y
2. Was the study population clearly specified and defined?	Y	Y	Y	Y	Y	Y	Y	Y	Y
3. Was the participation rate of eligible persons at least 50%?	CD	Y	CD	CD	Y	CD	CD	CD	CD
4. Were all the subjects selected or recruited from the same or similar populations (including the same time period)? Were inclusion and exclusion criteria for being in the study prespecified and applied uniformly to all participants?	Y	Y	Y	Y	Y	Y	CD	Y	Y
5. Was a sample size justification, power description, or variance and effect estimates provided?	N	N	Y	N	Y	Y	Y	Y	N
6. For the analyses in this paper, were the exposure(s) of interest measured prior to the outcome(s) being measured?	Y	Y	Y	Y	Y	Y	Y	Y	Y
7. Was the timeframe sufficient so that one could reasonably expect to see an association between exposure and outcome if it existed?	Y	Y	Y	Y	Y	Y	Y	Y	Y
8. For exposures that can vary in amount or level, did the study examine different levels of the exposure as related to the outcome (e.g., categories of exposure, or exposure measured as continuous variable)?	N	N	Y	N	N	N	N	N	Y
9. Were the exposure measures (independent variables) clearly defined, valid, reliable, and implemented consistently across all study participants?	Y	N	Y	Y	Y	Y	Y	Y	Y
10. Was the exposure(s) assessed more than once over time?	NA	NA	NA	NA	NA	NA	NA	NA	NA
11. Were the outcome measures (dependent variables) clearly defined, valid, reliable, and implemented consistently across all study participants?	N	Y	Y	N	N	N	N	Y	Y

12. Were the outcome assessors blinded to the exposure status of participants?	N	N	N	N	N	N	N	N	N
13. Was loss to follow-up after baseline 20% or less?	Y	Y	Y	Y	Y	Y	Y	Y	Y
14. Were key potential confounding variables measured and adjusted statistically for their impact on the relationship between exposure(s) and outcome(s)?	Y	N	Y	N	N	Y	Y	Y	N
Total percentage of applicable questions answered 'Yes'	**57.1%**	**57.1%**	**78.6%**	**50%**	**64.3%**	**64.3%**	**57.1%**	**71.4%**	**64.3%**

Table 2. Quality Assessment Tool for Observational Cohort and Cross-Sectional Studies. CD, Cannot determine; N/R, Not recorded; N/A, Not applicable.

Criteria	Gobbi et al. 2014	Gobbi et al. 2005	Ollat et al.	Gudas et al.	Viste et al.	Pelissier et al.	Minzlaff et al.	Pestka et al.	Stone et al.
1. Was the research question or objective in this paper clearly stated?	Y	Y	Y	Y	Y	Y	Y	Y	Y
2. Was the study population clearly specified and defined?	Y	Y	Y	Y	Y	Y	Y	Y	Y
3. Was the participation rate of eligible persons at least 50%?	Y	N	Y	Y	CD	CD	Y	Y	Y
4. Were all the subjects selected or recruited from the same or similar populations (including the same time period)? Were inclusion and exclusion criteria for being in the study prespecified and applied uniformly to all participants?	Y	Y	Y	Y	Y	Y	Y	Y	Y
5. Was a sample size justification, power description, or variance and effect estimates provided?	N	N	N	Y	N	Y	Y	N	Y
6. For the analyses in this paper, were the exposure(s) of interest measured prior to the outcome(s) being measured?	Y	Y	Y	Y	Y	Y	Y	N	N
7. Was the timeframe sufficient so that one could reasonably expect to see an association between exposure and outcome if it existed?	Y	Y	Y	Y	Y	Y	Y	Y	Y
8. For exposures that can vary in amount or level, did the study examine different levels of the exposure as related to the outcome (e.g., categories of exposure, or exposure measured as continuous variable)?	N	Y	N	N	N	N	N	N	N
9. Were the exposure measures (independent variables) clearly defined, valid, reliable, and implemented consistently across all study participants?	Y	Y	N	Y	Y	Y	Y	Y	Y
10. Was the exposure(s) assessed more than once over time?	NA	NA	NA	NA	NA	NA	NA	NA	N
11. Were the outcome measures (dependent variables) clearly defined, valid, reliable, and implemented consistently across all study participants?	Y	N	N	Y	N	Y	Y	Y	N

12. Were the outcome assessors blinded to the exposure status of participants?	N	N	N	Y	N	N	N	N	N
13. Was loss to follow-up after baseline 20% or less?	Y	Y	Y	Y	Y	Y	Y	Y	Y
14. Were key potential confounding variables measured and adjusted statistically for their impact on the relationship between exposure(s) and outcome(s)?	Y	Y	N	Y	N	N	Y	Y	N
Total percentage of applicable questions answered ‘Yes’	**71.4%**	**64.3%**	**50%**	**85.7%**	**50%**	**64.3%**	**78.6%**	**64.3%**	**57.1%**

Criteria	Nielsen et al.	McCarthy et al.	Cotter et al.	Zaffagnini et al.	Monckeberg et al.	Liu et al.	Scillia et al.	Tetta et al.	Ronga et al.
1. Was the research question or objective in this paper clearly stated?	Y	Y	Y	Y	Y	Y	Y	Y	Y
2. Was the study population clearly specified and defined?	Y	Y	Y	Y	Y	Y	Y	Y	Y
3. Was the participation rate of eligible persons at least 50%?	Y	Y	Y	CD	CD	Y	Y	CD	CD
4. Were all the subjects selected or recruited from the same or similar populations (including the same time period)? Were inclusion and exclusion criteria for being in the study prespecified and applied uniformly to all participants?	Y	Y	Y	Y	Y	Y	Y	Y	Y
5. Was a sample size justification, power description, or variance and effect estimates provided?	N	N	N	N	N	N	N	Y	N
6. For the analyses in this paper, were the exposure(s) of interest measured prior to the outcome(s) being measured?	N	Y	N	Y	Y	N	N	Y	Y
7. Was the timeframe sufficient so that one could reasonably expect to see an association between exposure and outcome if it existed?	Y	Y	Y	Y	Y	Y	Y	Y	Y
8. For exposures that can vary in amount or level, did the study examine different levels of the exposure as related to the outcome (e.g., categories of exposure, or exposure measured as continuous variable)?	N	N	N	N	N	N	Y	Y	N
9. Were the exposure measures (independent variables) clearly defined, valid, reliable, and implemented consistently across all study participants?	Y	Y	Y	Y	Y	Y	N	Y	Y
10. Was the exposure(s) assessed more than once over time?	NA	NA	NA	NA	NA	NA	NA	NA	NA
11. Were the outcome measures (dependent variables) clearly defined, valid, reliable, and implemented consistently across all study participants?	N	Y	N	Y	N	N	Y	Y	N

12. Were the outcome assessors blinded to the exposure status of participants?	N	N	N	N	N	N	N	N	N
13. Was loss to follow-up after baseline 20% or less?	Y	Y	Y	Y	Y	N	Y	Y	Y
14. Were key potential confounding variables measured and adjusted statistically for their impact on the relationship between exposure(s) and outcome(s)?	N	N	Y	Y	N	N	Y	Y	N
Total percentage of applicable questions answered 'Yes'	**50%**	**64.3%**	**57.1%**	**64.3%**	**50%**	**42.9%**	**57.1%**	**78.6%**	**50%**

**Criteria**	**Vijayan et al.**	**Gillogly et al.**
1. Was the research question or objective in this paper clearly stated?	Y	Y
2. Was the study population clearly specified and defined?	Y	Y
3. Was the participation rate of eligible persons at least 50%?	Y	CD
4. Were all the subjects selected or recruited from the same or similar populations (including the same time period)? Were inclusion and exclusion criteria for being in the study prespecified and applied uniformly to all participants?	Y	Y
5. Was a sample size justification, power description, or variance and effect estimates provided?	N	N
6. For the analyses in this paper, were the exposure(s) of interest measured prior to the outcome(s) being measured?	Y	Y
7. Was the timeframe sufficient so that one could reasonably expect to see an association between exposure and outcome if it existed?	Y	Y
8. For exposures that can vary in amount or level, did the study examine different levels of the exposure as related to the outcome (e.g., categories of exposure, or exposure measured as continuous variable)?	N	N
9. Were the exposure measures (independent variables) clearly defined, valid, reliable, and implemented consistently across all study participants?	Y	Y
10. Was the exposure(s) assessed more than once over time?	N	N
11. Were the outcome measures (dependent variables) clearly defined, valid, reliable, and implemented consistently across all study participants?	N	N

12. Were the outcome assessors blinded to the exposure status of participants?	N	N
13. Was loss to follow-up after baseline 20% or less?	Y	Y
14. Were key potential confounding variables measured and adjusted statistically for their impact on the relationship between exposure(s) and outcome(s)?	N	N
Total percentage of applicable questions answered ‘Yes’	**57.1%**	**50%**
